# Exosomal miR-483-5p in Bone Marrow Mesenchymal Stem Cells Promotes Malignant Progression of Multiple Myeloma by Targeting TIMP2

**DOI:** 10.3389/fcell.2022.862524

**Published:** 2022-03-01

**Authors:** Jianmei Gu, Maoye Wang, Xinfeng Wang, Jiao Li, Haiyan Liu, Zenghua Lin, Xi Yang, Xu Zhang, Hong Liu

**Affiliations:** ^1^ Department of Hematology, First Affiliated Hospital of Soochow University, Suzhou, China; ^2^ Department of Clinical Laboratory Medicine, Affiliated Cancer Hospital of Nantong University, Nantong, China; ^3^ Jiangsu Key Laboratory of Medical Science and Laboratory Medicine, School of Medicine, Jiangsu University, Zhenjiang, China; ^4^ Department of Hematology, Affiliated Hospital of Nantong University, Nantong, China

**Keywords:** multiple myeloma, BMSCs, exosomes, miR-483-5p, TIMP2

## Abstract

Bone marrow-derived mesenchymal stem cell (BMSC) is one crucial component of the multiple myeloma (MM) microenvironment and supports the malignant progression of MM. Whether BMSCs act on MM cells via exosomes has not been well characterized. Herein, we used microarrays to screen out differentially expressed miRNAs in BMSCs from patients with MM (MM-MSCs) or benign diseases (BD-MSCs). We found that miR-483-5p was highly expressed in MM-MSCs, which may be transported through exosomes from MM-MSCs to MM cells to increase miR-483-5p expression in them. We then investigated the role and mechanism of miR-483-5p in the aggressive progression of MM *in vitro*. We verified that miR-483-5p promoted MM cell proliferation and reduced apoptosis. Then we predicted and validated that TIMP2, a tumor suppressor gene, is the downstream target of miR-483-5p in MM. In summary, our study indicated that MM-MSCs promote MM malignant progression via the release of exosomes and regulation of miR-483-5p/TIMP2 axis, suggesting an essential role of BMSCs derived exosomal miRNA in MM and a potential marker for MM diagnosis and therapy.

## Introduction

Multiple myeloma (MM) is the second most common hematological malignancy after non-Hodgkin lymphoma (Siegel et al., 2021). MM is a diffuse neoplasm of bone marrow B cell-derived plasma cells (PCs) and the malignant cells mingle with the hematopoietic cells throughout the bone marrow (BM) (Palumbo and Anderson, 2011). Though emerging therapies such as immunotherapy and targeted therapeutics have evolved, MM remains an incurable disease and causes end-organ damage, including hypercalcemia, renal impairment, anemia and bone lesions (Shah and Mailankody, 2020). Therefore, understanding the molecular mechanism of MM will be critical to prolonging the survival of MM patients.

The bone marrow microenvironment consists of different types of cells, and bone marrow mesenchymal stem cell (BMSC) is the leading stem cell type in human bone marrow (Weinberg, 2008; Zahn, 2017; Dart, 2018; Nwabo Kamdje et al., 2020). BMSCs play an essential role in the malignant progression of hematologic diseases, including MM (Jin et al., 2019; Lee et al., 2019; Liu et al., 2021). BMSCs interact with other cells by cell-to-cell contacts and secretion of soluble factors. For example, studies have shown that MM cell growth and phenotype may change when co-cultured with BMSC cells (Fuhler et al., 2010). Besides, BMSCs have been reported to secrete excessive interleukin-6 (IL-6) to promote and support the growth of MM cells (Arnulf et al., 2007). In addition, another study has revealed that BMSCs could protect MM cells from destruction by reactive cytotoxic T cells (CTL) and NK cells, triggering immune escape (Giannakoulas et al., 2021). In turn, MM cells could also educate and manipulate BMSCs to make a more conducive microenvironment for MM cell growth. For instance, recent studies have demonstrated that co-culturing normal BMSCs with MM cells could inhibit osteoblastic differentiation of BMSCs, which aggravates the progression of MM (Mehdi et al., 2019).

Increasing studies have suggested that in addition to direct cell-to-cell contact and cytokines, exosomes are considered as another important mediator of intercellular material exchange (Colombo et al., 2014; Lo Cicero et al., 2015; Willms et al., 2016). Exosomes are nanoscale extracellular vesicles actively secreted by cells, enriched with nucleic acids, proteins, lipids, metabolites and even organelles. The lipid bilayer membranes of exosomes protect their carriers from degradation in the circulation, making exosomes a key mediator of tumor-stroma communication (O'Brien et al., 2020). It has been reported that exosomal miR-7-5p derived from BMSCs could negatively regulate OSBPL11 via PI3K/AKT/mTOR signaling pathway in acute myeloid leukemia (AML) and finally inhibit AML proliferation and promote apoptosis (Jiang et al., 2022). Besides, exosomes released from chronic myelogenous leukemia (CML) cells could stimulate BMSCs to produce IL-8, promoting CML cells malignant phenotype (Corrado et al., 2014). Recently, Wang et al. have identified that adipocyte exosomal lncRNA LOC606724 and SNHG1 may contribute to bortezomib resistance in MM cells (Wang et al., 2022). These studies suggest that exosomes participate in the progression of many hematologic malignancies and may be a potential target for them.

Up to now, studies on the regulation of MM cells by exosomes from MM-MSCs are limited. To understand the association between MM and exosomes from MM-MSCs, we used the gene microarray to detect the abnormally expressed miRNAs in MM-MSCs. We hypothesized that miRNAs carried by exosomes of MM-MSCs and delivered to MM cells might promote the malignant progression of MM cells. Our study will help gain a better understanding of the biological role of BMSCs and their exosomes in MM progression.

## Materials and Methods

### Isolation of BMSCs From MM Patients

All eight patients with MM were admitted to the Affiliated Hospital of Nantong University from September 2016 to December 2019, and the diagnostic criteria and treatment efficacy were referenced to the International Myeloma Working Group. All the cases included in this study were primary and untreated MM patients, with a mean age of 66 years (50–70 years). In the control group, 5 cases with a mean age of 62 years (50–70 years) were selected from patients with benign diseases (BD), including iron deficiency anemia and thrombocytopenia. The collection and use of clinical specimens were approved by the ethics committee of Affiliated Hospital of Nantong University with written informed consent. BM samples from newly diagnosed MM and BD patients were harvested from their iliac bones. Total 3–4 ml of BM was collected and placed into EDTA anticoagulant tubes. All procedures were carried out under sterile conditions.

BM mononuclear cells were isolated using human BM mononuclear lymphocytes separating medium (Biological Products Technology; Tianjin Haoyang; China). In brief, BM was added slowly into the separating medium and then centrifuged at 1,000 × g for 20 min at 20°C. Slow acceleration and deceleration settings were used. Finally, primary BMSCs were cultured and selected in plastic flasks by adherence method and used at the third to fourth passage. The MM cell lines RPMI 8226 and U266 used in *in vitro* experiments were purchased from the Cell Bank of the Chinese Academy of Sciences (Shanghai, China).

### Phenotype Identification of BMSCs

Flow cytometry was used to identify phenotypic surface markers of BMSCs with antibodies, including PE-conjugated anti-CD29, anti-CD73, anti-CD105, anti-CD11b, anti-CD34 and anti-CD45 antibodies (Cyagen, China). Appropriate isotype controls were used. Data analysis was performed on FlowJo 10.6.2 (FlowJo, LLC).

### Osteogenic and Lipogenic Differentiation of BMSCs

The osteogenic differentiation of BMSCs was performed using the osteogenic differentiation medium kit (HUXMA-90021, Cyagen, China). The osteogenic differentiation complete medium was prepared according to the kit instructions and culture medium was changed every 3 days. Finally, the osteogenic differentiation was performed and analyzed by alizarin red staining after the induction of differentiation.

Lipogenic differentiation of BMSCs was performed using the lipogenic differentiation medium kit (HUXMA-90031, Cyagen, China). Lipogenesis-induced differentiation A and B solutions were prepared according to the kit instructions and induced until large and round lipid droplets appeared. Then, Oil Red O was used to detect lipid droplets.

### Gene Microarray

Total RNA was extracted from BMSCs isolated from MM and BD patients. RNA was quantified using a NanoDrop ND-2000 (Thermo Fisher Scientific, Waltham, MA, United States), and RNA integrity was assessed using Agilent Bioanalyzer 2100 (Agilent Technologies, Santa Clara, CA, United States). After RNA qualification, sample labeling, microarray hybridization, and washing were performed according to the standard process. First, total RNA was transcribed to double-stranded cDNA and then Cyanine-3-CTP(Cy3)-labeled cRNA was synthesized; the labeled cRNA was hybridized to the microarray [miRNA: Agilent Human miRNA Microarray Kit, release 21.0,8 × 60K (DesignID:070,156)]. The labeled cRNA was hybridized to the chip, and the original image was scanned by Agilent Scanner G2505C (Agilent Technologies) after elution. After washing, the arrays were scanned using a G2505C scanner (Agilent Technologies).

Microarray analysis was performed by OE Biotech (Shanghai, China). Feature Extraction software (version10.7.1.1, Agilent Technologies) was used to analyze array images to get raw data. GeneSpring (version 14.8, Agilent Technologies) was employed to finish the basic analysis with the raw data. At first, the raw data were normalized with the quantile algorithm. The probes that at least one out of two conditions have flags in ‘Detected’ were chosen for further data analysis. Differentially expressed genes were then identified through fold change as well as *p* value calculated with *t*-test. The threshold set for up-and down-regulated genes was a fold change ≥1.5 and a p-value ≤ 0.05. Afterward, GO and KEGG analyses were applied to determine the roles of these differentially expressed mRNAs. Finally, Hierarchical Clustering was performed to display the differentially expressed genes’ expression pattern among samples.

### RNA Extraction and Quantitative Real-Time PCR

Total RNA was extracted with Trizol reagent (Invitrogen, CA, United States), and the cDNA was synthesized using a reverse transcription kit (Ribobio, China or Vazyme, Nanjing, China) according to the manufacturer’s instructions. miRNA-related primers were produced by Ribobio Company. The sequences of β-Actin primers were as follows: forward, 5′-CAC​GAA​ACT​ACC​TTC​AAC​TCC-3’; reverse, 5′-CAT​ACT​CCT​GCT​TGC​TGA​TC-3′. The sequences of TIMP2 primers were as follows: forward, 5′-GCT​GCG​AGT​GCA​AGA​TCA​C-3′; reverse, 5′-TGG​TGC​CCG​TTG​ATG​TTC​TTC-3′. We used quantitative PCR to detect the expression of miR-483-5p (miDETECT A TrackTM, Ribobio, China) and TIMP2 (Vazyme, Nanjing, China). U6 snRNA or β-Actin were used as internal controls. All samples were run in triplicate, and all reactions were repeated three times independently to ensure reproducibility.

### Isolation of Exosomes From BMSCs

Exosomes were isolated using the ultracentrifugation method. In brief, cells were grown in α-MEM medium supplemented with 10% exosome-depleted FBS. After 48 h, the culture medium was collected and subjected to sequential centrifugation at 300 × g for 20 min, 2,000 × g for 20 min, and 10,000 × g for 30 min at 4°C to remove residual cells, debris and organelles. Then, ultracentrifugation at 100,000 × g for 2 h was used to collect exosomes.

### Gene Silencing and Overexpression

miR-483-5p inhibitor (5′-CCC​UCC​ACC​AUG​CAA​GGG​AUG-3′), inhibitor negative control (5′-CUC​CCU​UCU​UUC​CUC​CCG​UCU​U-3′), miR-483-5p mimics (sense, 5′-AAG​ACG​GGA​GGA​AAG​AGG​GAG-3’; antisense, 5′-CCC​UUC​UUU​CCU​CCC​GUC​UUU​U-3′) and mimic negative control (sense, 5′-UUC​UCC​GAA​CGU​GUC​ACG​UTT-3’; antisense, 5′-ACG​UGA​CAC​GUU​CGG​AGA​ATT-3′) (GenePharma, Shanghai, China) were transfected into the cells by using lipofectamine 2000 (Invitrogen, CA, United States) in serum-free medium. After 6 h of incubation, the medium was changed to complete medium.

### Cell Apoptosis Assay

Cell apoptosis was detected by flow cytometry using Annexin V-Alexa Fluor 647/PI apoptosis detection kit (FcMACS, Nanjing, China). The transfected cells were washed twice with pre-cooled PBS at 4°C and resuspended with the binding buffer and 100 μL of cell suspension (1×10^6^/ml) was incubated with 5 μL of Annexin V-Alexa Fluor 647 and 10 μL of 20 μg/ml of propidium iodide solution at room temperature for 15 min. Finally, 400 μL of PBS was added to the reaction tube and the cells were analyzed by flow cytometry (Beckman Coulter, United States).

### Dual-Luciferase Reporter Assay

Targetscan (http://www.targetscan.org/vert_80/), ENCORI (https://starbase.sysu.edu.cn/), miRDB (http://mirdb.org/) and miRwalk (http://mirwalk.umm.uni-heidelberg.de/) were used to predict the potential target mRNAs of miR-483-5p. RPMI 8226 and U266 cells were co-transfected with miR-483-5p mimics and the luciferase reporter vector containing wild type (WT) 3′-UTR of TIMP2 (GenePharma, Shanghai, China) by lipofectamine 2000. The medium was changed after 6 h of transfection and cells were lysed after 36 h of incubation. At last, luciferase activity was detected by the Reporter Assay Program Dual-Luciferase (Promega).

### Statistical Analysis

All data were expressed as means ± SD. All statistical analysis was performed using IBM SPSS 21.0 (SPSS, Chicago, IL, United States) or Graph Pad Prism 7.0 (Graph Pad Software, Inc. San Diego, CA, United States). All reported p-values are two-tailed, and *p* < 0.05 was considered statistically significant.

## Results

### Characterization of BMSCs

BMSCs were isolated by density gradient centrifugation and adherence method. Then we identified BMSCs from BD and MM patients by cell morphology, surface markers and differentiation ability.

**FIGURE 1:**
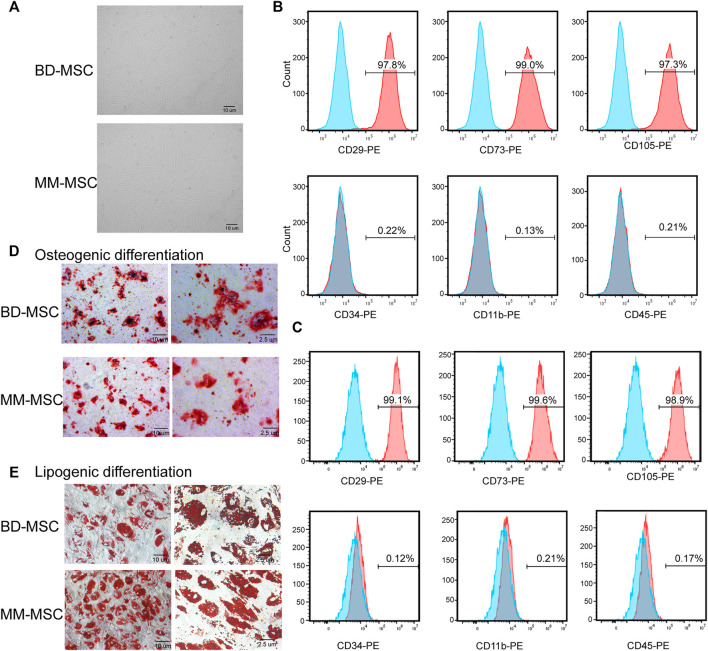
Identification of BMSCs from patients with BD and MM. **(A)** Morphologies of BMSCs from BD or MM patients were determined using light microscopy. BMSCs grew with increased adherence and exhibited a long spindle shape. **(B,C)** Surface markers of BD-MSCs **(B)** or MM-MSCs **(C)** were detected by flow cytometry. Cells were CD29, CD73, CD105 positive and CD34, CD11b, CD45 negative. **(D)** Osteogenic differentiation of BMSCs from BD or MM patients. **(E)** Lipogenic differentiation of BMSCs from BD or MM patients.

Microscopic observations showed that the cell morphology of BD-MSCs and MM-MSCs were similar, both showing long fiber-like spindle shapes (Figure 1A). Flow cytometry results showed that BMSCs positively expressed CD29, CD73, CD105 (>97%), while negatively expressed CD45, CD34, and CD11b (<0.3%) (Figures 1B,C). All results showed that these cells contain a characteristic set of surface markers of BMSCs (Mushahary et al., 2018).

Osteogenesis induction results showed that primary cultured BMSCs formed small lamellar mineralized nodules after 21 days of culture, but the bone nodules from BD-MSCs were darker and larger in appearance compared to those from MM-MSCs (Figure 1D). The results of lipogenesis induction showed that primary cultured BMSCs were easily induced to differentiate into adipogenic direction after 21 days. After Oil Red O staining, a large number of granular red lipid droplets in the cytoplasm were shown. Moreover, MM-MSCs had more lipid droplets (Figure 1E). Therefore, we speculated that the osteogenic capacity of MSCs in MM patients was reduced while their lipogenic capacity was enhanced.

### Distinct Gene Expression Profiling, Network and Functional Enrichment Analyses

**FIGURE 2:**
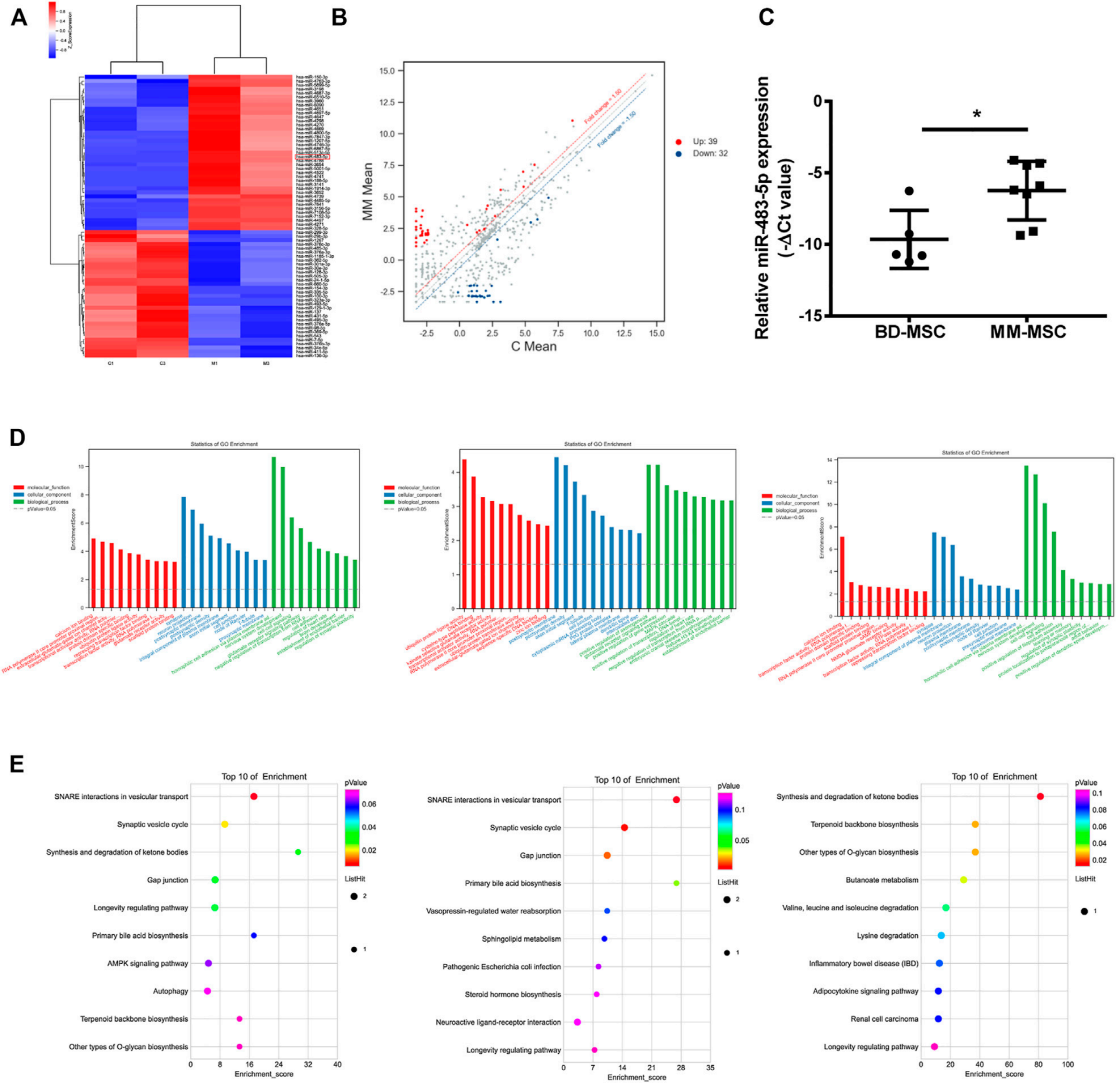
Distinct gene expression profiling, network and functional enrichment analyses. **(A)** Cluster plots showed the expression of miRNAs in BMSCs from MM and BD patients. **(B)** Scatter plots showed gene expression changes in MM-MSCs and BD-MSCs. **(C)** miR-483-5p expression was validated by qRT-PCR. **(D)** GO enrichment analysis. **(E)** KEGG enrichment analysis.

To explore the differences between BMSCs of MM and BD patients, We selected two paired BD-BMSCs and MM-BMSCs for differential gene screening by gene microarray, and the screening criteria were up-regulated or down-regulated fold change value ≥ 1.5 and p-value ≤0.05. Cluster and scatter plots demonstrated the differentially expressed miRNAs among them (Figures 2A,B). The clustered map showed 39 up-regulated miRNAs, including miR-483-5p and 32 down-regulated ones in MM-MSCs compared to BD-MSCs (Figure 2A). qPCR verification showed that miR-483-5p was up-regulated in BMSCs from MM patients compared to BD patients (Figure 2C).

GO enrichment analysis bar graphs showed the entries of differential gene-related biological processes (BP), cellular components (CC), and molecular functions (MF). The different color distributions correspond to BP, CC, and MF. We performed enrichment analyses for all, up-regulated, and down-regulated differential genes for BP, CC, and MF, respectively. The top 10 most significant GO entries in the three major GO categories were shown (Figure 2D).

KEGG enrichment analysis was performed to explore pathways associated with differential genes. This KEGG enrichment analysis was performed separately for all, up-regulated, and down-regulated differential genes (Figure 2E). KEGG enrichment analysis explored the signaling pathways that might be associated with the differential genes (Figure 2E).

### MM-MSC-Ex Promotes MM Cell Proliferation

**FIGURE 3:**
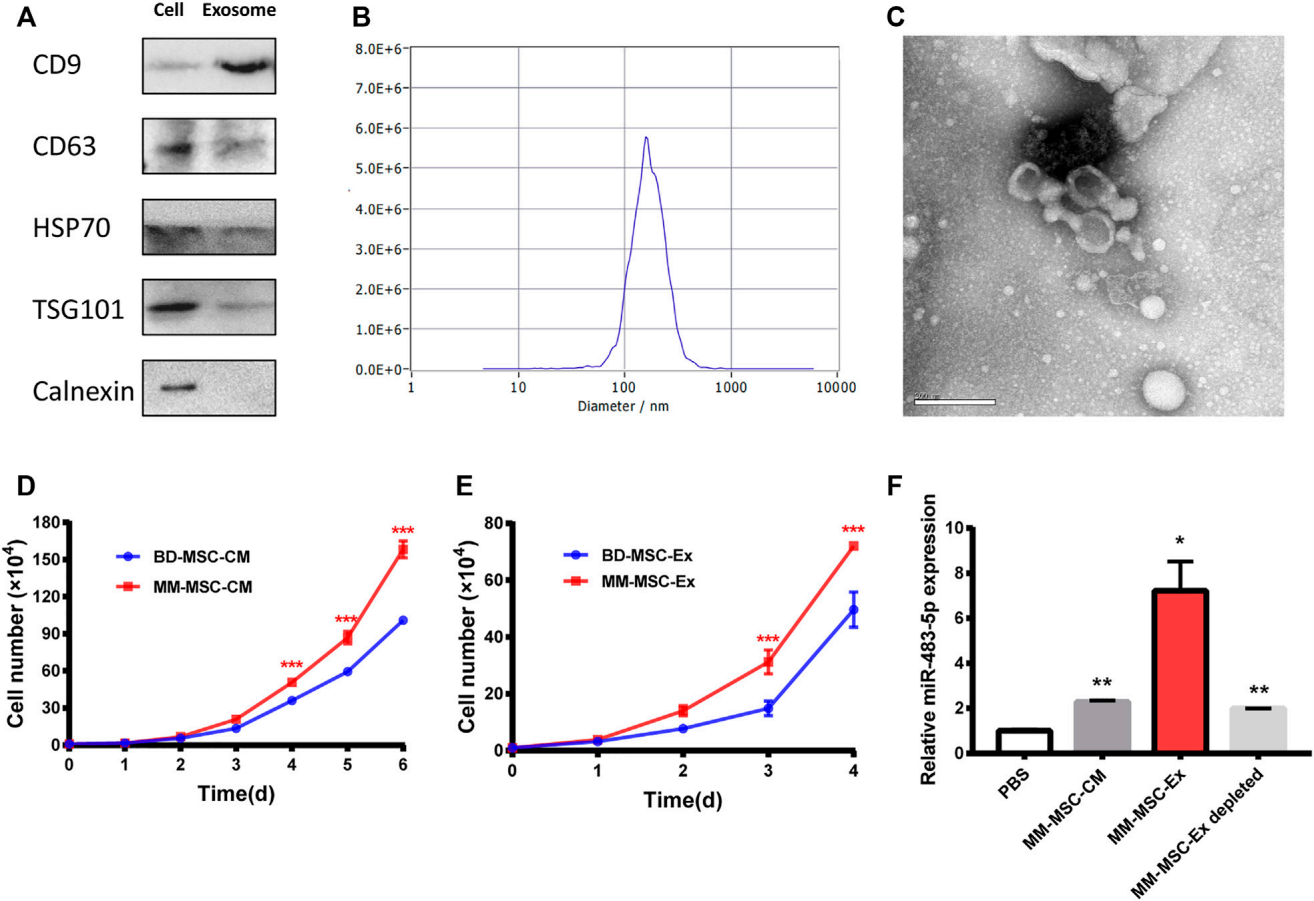
Exosome characterization and function. **(A)** Exosome-related positive-markers like CD9, CD63, HSP70, TSG101 and negative-markers like Calnexin were detected by Western blot **(B)** The particle size was determined by Nanosight particle analysis. **(C)** TEM analysis showed the cup-shaped morphology of exosomes. **(D, E)** Both MM-MSC conditioned medium **(D)** and exosomes **(E)** showed significant pro-proliferative roles in MM cells compared with BD-MSCs. **(F)** miR-483-5p expression after treatment with conditioned medium or exosomes was tested by qRT-PCR.

Exosomes act as intermediaries for intercellular communication. Exosomes from BMSCs were isolated by ultracentrifugation and characterized by Western blot (Figure 3A), NTA (Figure 3B) and TEM (Figure 3C). Interestingly, MM cells treated with conditioned medium (CM) from MM-MSCs showed an enhanced proliferation ability than those treated with CM from BD-MSCs (Figure 3D). We also observed a similar effect in MM cells treated with exosomes from MM-MSCs (Figure 3E). Furthermore, MM-MSC-Ex treatment significantly increased miR-483-5p expression in MM cells (Figure 3F).

### miR-483-5p Promotes MM Aggressive Progression

**FIGURE 4:**
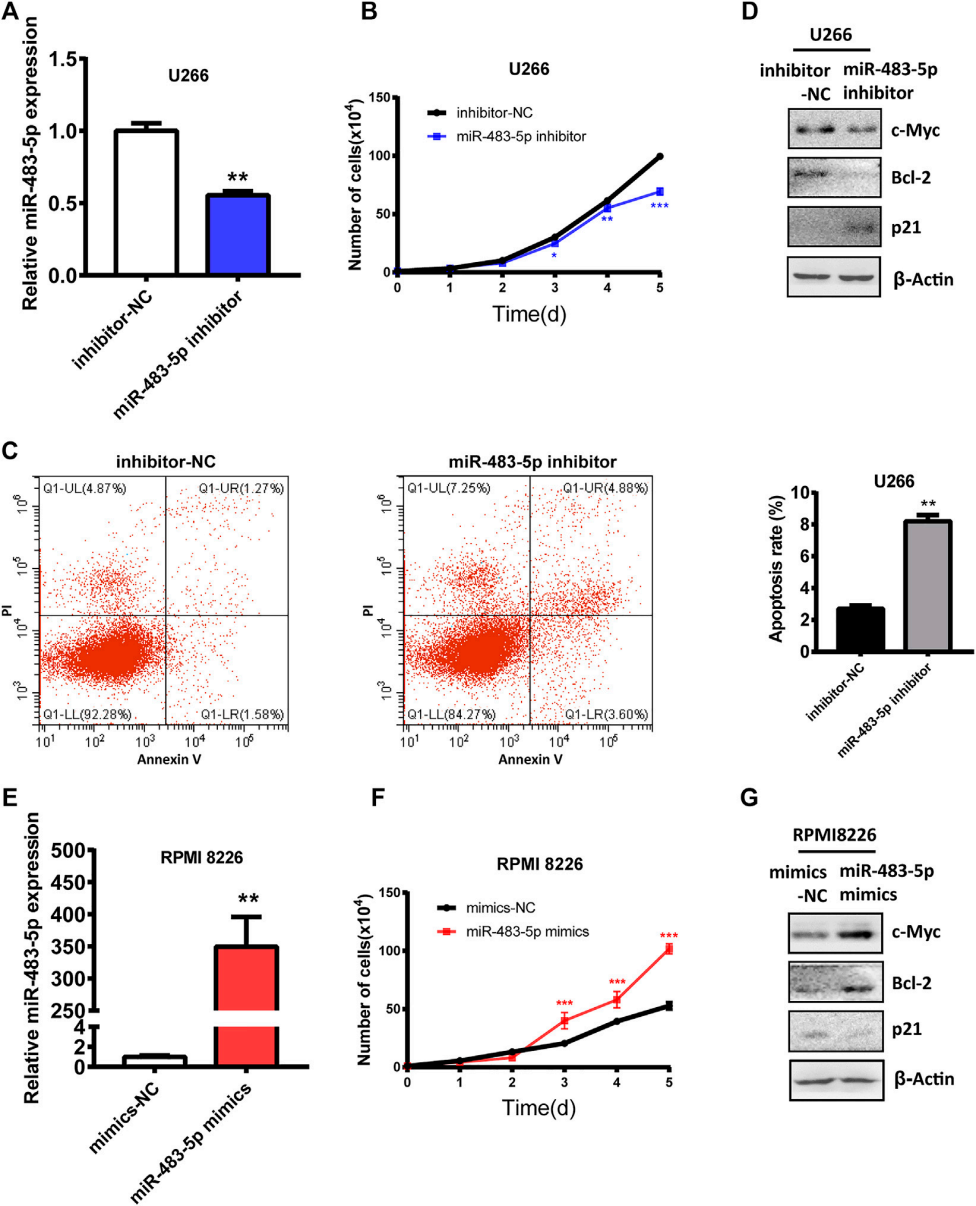
The functional role of miR-483-5p in MM cells. **(A)**The inhibition efficiency was verified by qRT-PCR after transfection of the miR-483-5p inhibitor. **(B–D)** The cell curve analysis **(B)**, flow cytometry analysis **(C)** and Western blot results **(D)** showed the decreased proliferation ability and increased apoptosis of U266 after transfection with miR-483-5p inhibitor. **(E)** qRT-PCR was used to detect the efficiency of RPMI 8226 transfection of miR-483-5p mimics. **(F,G)** The cell curve analysis **(F)** and Western blot results **(G)** revealed the enhanced proliferation ability of RPMI 8226 after transfection with miR-483-5p mimics.

To explore the role and mechanism of miR-483-5p in the malignant progression of MM cells, we transfected miR-483-5p inhibitor into U266 cells, which expressed higher miR-483-5p than RPMI 8226 (Supplementary Figure S1). First, we examined the transfection efficiency of miR-483-5p inhibitor, and we found that miR-483-5p level was significantly decreased after transfection (Figure 4A). In addition, cell proliferation ability of U266 was impaired after transfection (Figure 4B), while cell apoptosis was promoted (Figure 4C). In accordance, Western blot results revealed that miR-483-5p inhibitor resulted in a down-regulated level of c-Myc, Bcl-2, and up-regulated p21 expression in MM cells (Figure 4D), which are markers of cell proliferation and apoptosis. In contrast, the opposite results were observed with transfection of miR-483-5p mimics in RPMI 8226 cell line (Figures 4E–G). In summary, miR-483-5p impacted MM cell proliferation and apoptosis, thereby accelerating the malignant process of MM.

### TIMP2 Is the Target of miR-483-5p

**FIGURE 5:**
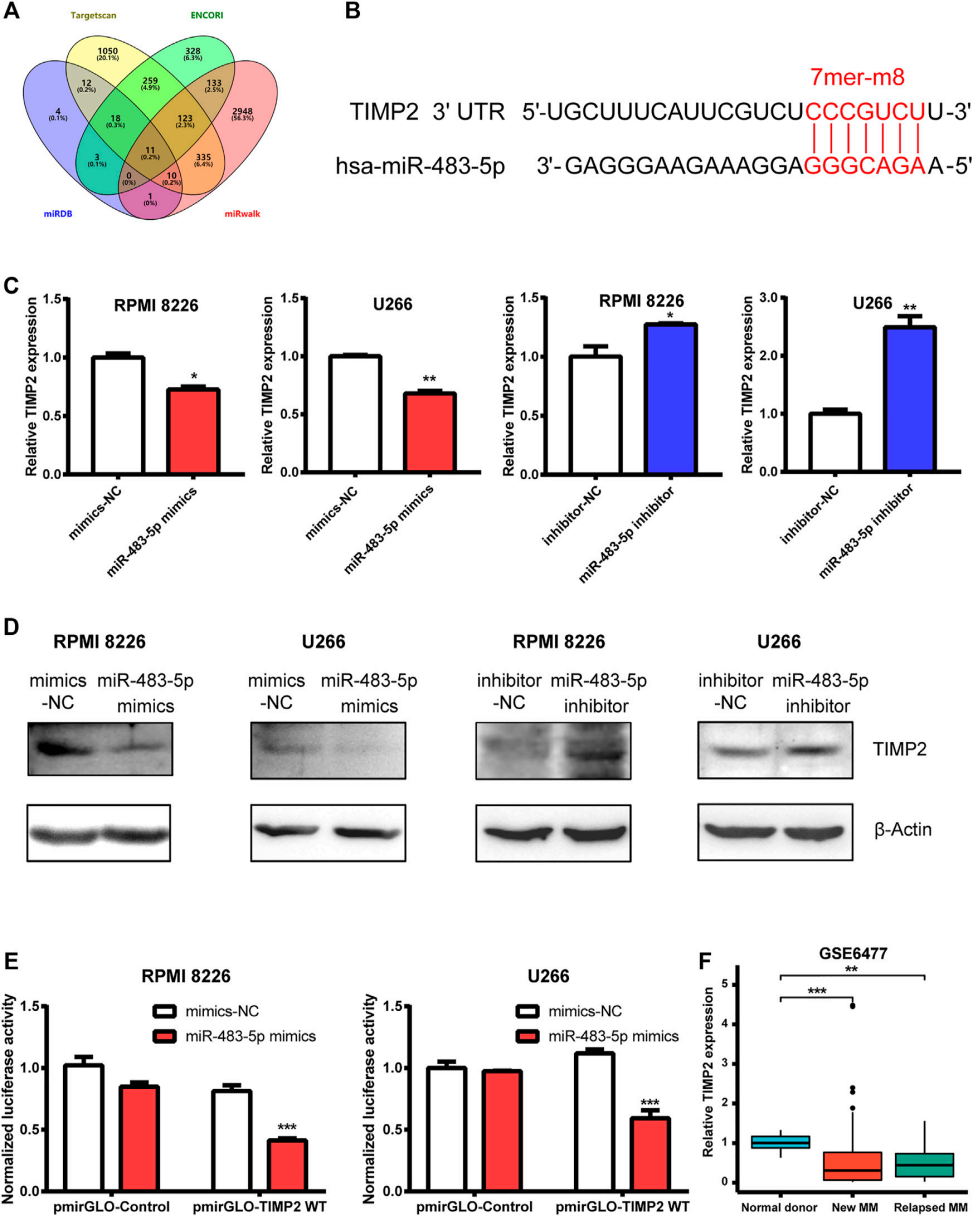
TIMP2 is one downstream target of miR-483-5p. **(A)** Venn diagram revealed the predicted target genes of miR-483-5p from four bioinformatic tools. **(B)** Bioinformatic prediction of the binding sites in miR-483-5p and TIMP2. **(C,D)** TIMP2 mRNA **(C)** and protein **(D)** expression were decreased in MM cells transfected by miR-483-5p mimics while increased when transfected by miR-483-5p inhibitor. **(E)** The dual-luciferase assay showed that miR-483-5p decreased the luciferase activity of WT TIMP2 luciferase reporter. **(F)** TIMP2 mRNA level was down-regulated in MM patients compared with normal subjects.

**FIGURE 6:**
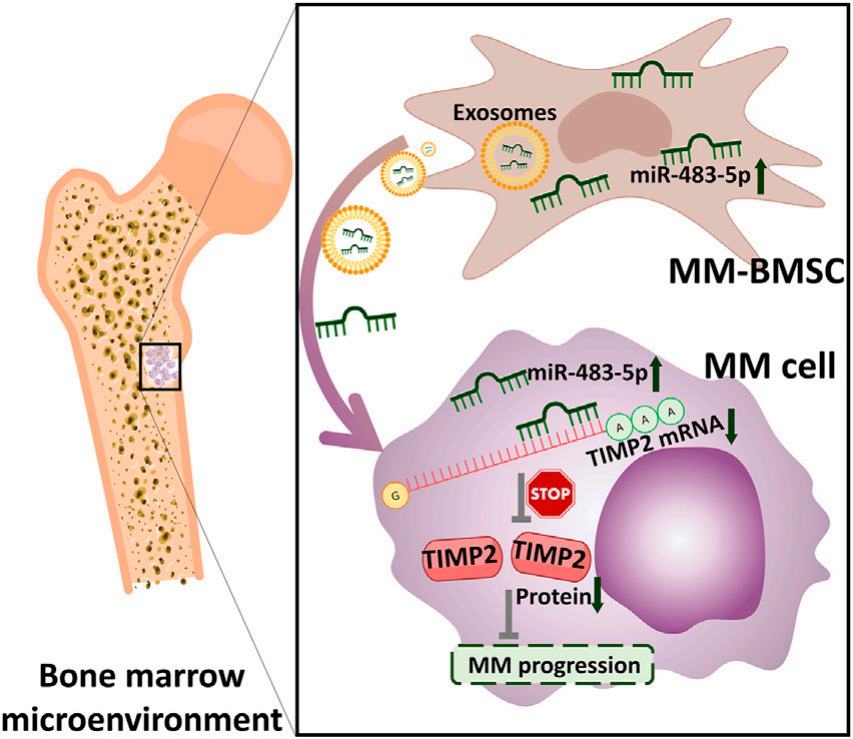
Proposed model for the mechanism of action of miR-483-5p in MM.

To search for a potential downstream target of miR-483-5p, we used miRNA prediction programs including Targetscan, ENCORI, miRDB and miRwalk as bioinformatics methods. TIMP2 was one intersection among them (Figure 5A). miR-483-5p and TIMP2 contained a perfect 7mer-m8 seeding match (Figure 5B). To detect their relation, we found that TIMP2 mRNA and protein expression was negatively correlated with miR-483-5p, tested by qRT-PCR and Western blot, respectively (Figures 5C,D). To further verify the relationship between miR-483-5p and TIMP2, the TIMP2 luciferase reporter plasmid was co-transfected with miR-483-5p mimics into MM cells. Compared to nective control, co-transfection with pmirGLO-TIMP2 WT and miR-483-5p mimics suppressed the luciferase activity by around 50% (Figure 5E). We speculated that miR-483-5p might promote MM progression by targeting TIMP2. For further analysis, we downloaded the GSE6477 dataset from GEO database, and we found that TIMP2 expression was lower in both newly diagnosed and relapsed MM patients than normal donors, which was consistent with our expectation. These results suggested that miR-483-5p/TIMP2 axis may play a vital role in MM (Figure 6).

## Discussion

BMSCs, as important cells in the tumor microenvironment, play a vital role in contributing to the malignant progression of cancers, promoting immune escape, and even significantly affecting the tolerance to treatment and leading to relapse (Ridge et al., 2017). In our study, we isolated and cultured MM- or BD-MSCs and analyzed their differentially expressed miRNAs, hoping to provide new targets and strategies for diagnosing and treating MM. First, we isolated MM-MSCs and BD-MSCs. We found that their morphology and surface signatures were essentially similar, but MM-MSCs seem to have diminished osteogenic differentiation and enhanced lipogenic differentiation, indicating a delicate balance of adipogenic-osteogenic differentiation of BMSCs (Chen et al., 2016). The disruption of the osteogenic differentiation ability of BMSCs promotes the development of multiple myeloma bone disease (Giuliani et al., 2006). Then, we screened the differentially expressed miRNAs of MM-MSCs and BD-MSCs by gene microarray expression profiling. Consistent with gene microarray results, miR-483-5p was highly expressed in MM-MSCs by qRT-PCR.

Studies have shown that miR-483-5p was located at the 11p15.5 site of insulin-like growth factor 2 (IGF2) intron 2, and its expression was correlated with the expression of its host gene, IGF2 (Fu et al., 2005; Pepe et al., 2018). Besides, miR-483-5p has been found to promote cancer cell proliferation, metastasis, chemotherapy resistance, and decrease radiosensitivity in various cancers (Tian et al., 2019; Castro-Vega et al., 2020; Chen et al., 2020; Rattanapan et al., 2021). It has been shown that serum miR-483-5p might be an ideal biomarker for preoperative diagnosis, chemotherapy sensitivity and prognosis of tumors (Xu et al., 2021). Interestingly, previous research from Qu et al. has found that plasma miR-483-5p was up-regulated in MM patients than healthy controls. In addition, the area under the ROC curve was 0.745 and patients with higher miR-483-5p expression had shorter median progression-free survival, indicating miR-483-5p a potential diagnostic and predictor prognostic marker in MM (Qu et al., 2014). In our study, we knocked down the expression of miR-483-5p in U266, and MM cells showed reduced proliferation and increased apoptosis, while overexpression of miR-483-5p resulted in the opposite biological function of MM cells. Our findings suggested a similar role of miR-483-5p in MM as in other cancers, acting as a pro-oncogene promoting the malignant progression of MM. Interestingly, there was increasing evidence that miR-483-5p was involved in the pathogenesis of osteoporosis, and osteogenic differentiation and PI3K/AKT signaling were also regulated by the miR-483-5p/SATB2 axis (Zhao et al., 2021). We hypothesized that miR-483-5p was closely related to bone destruction as one of the clinical manifestations of MM. However, whether miR-483-5p could be a therapeutic target for the treatment of MM bone disease warrants further investigation.

Exosomes are secreted by almost all types of cells as an important type of extracellular vesicles. Exosomes carry a variety of biologically active molecules, such as nucleic acids and proteins. By carrying unique molecules, exosomes can play an essential role in the malignant progression of tumors as a vital mediator of cell-to-cell interactions. Our earlier work revealed that gastric cancer cells could induce differentiation of MSCs to CAFs by secreting exosomes, providing new evidence for the mode of action between tumor and tumor microenvironment cells (Gu et al., 2012). Recently, some studies have suggested that exosomes could also regulate crosstalk between BMSCs and MM cells, extending the communication method between them (Roccaro et al., 2013). It has been reported that young BMSC exosomes and their highly expressed exosomal miR-340 had greater antiangiogenic ability in MM cells compared to older BMSCs (Umezu et al., 2017). Another study indicated that exosomal miR-155 derived from BMSCs could facilitate MM cells’ resistance to chemotherapeutic drugs and maintain their stemness (Gao et al., 2021). Similarly, in our work, we found that exosomes from MM-MSCs act on MM cells by enhancing miR-483-5p level, thus promoting the malignant progression of MM. We then investigated the effect of miR-483-5p on the proliferation and apoptosis of MM cells by up- or down-regulating miR-483-5p expression, demonstrating that miR-483-5p played a pro-oncogene role in MM.

For further mechanism analysis, we predicted and verified that tissue inhibitor of metalloproteinase 2 (TIMP2) was one target gene of miR-483-5p. TIMP2 was a kind of matrix metalloproteinases (MMP) inhibitor, while MMPs were intriguing genes frequently up-regulated in most cancers (Gobin et al., 2019). Consistent with theoretical expectations, as an MMP inhibitor, TIMP2 was downregulated in many cancer types, and its high expression may indicate a better prognosis (Stetler-Stevenson and Gavil, 2014; Wang et al., 2021; Wang and Liu, 2021). In our research, we found that both TIMP2 mRNA and protein expression were negatively correlated with miR-483-5p. Furthermore, from the GSE6477 dataset, we also found that TIMP2 was down-regulated in newly diagnosed (*p* < 0.001) and relapsed MM patients (*p* < 0.01) when compared to normal donors. These results indicated miR-483-5p/TIMP2 axis might play an important role in MM progression.

In conclusion, we found that miR-483-5p was up-regulated in MM-MSCs and could be transferred from MM-MSCs to MM cells via exosomes to favor MM progression by targeting TIMP2. Our findings provided new evidence for the role of MM-MSCs in MM and a new mode of regulation for MM progression by exosomes. Nevertheless, our study also had some shortcomings. The controls were not completely healthy subjects. Most significantly, our sample size was relatively small.

## Data Availability

The datasets presented in this study can be found in online repositories. The names of the repository/repositories and accession number(s) can be found below: GEO accession GSE196321: Go to https://www.ncbi.nlm.nih.gov/geo/query/acc.cgi?acc=GSE196321.
