# Supplementary figures and images for: Exosomal miR-483-5p in Bone Marrow Mesenchymal Stem Cells Promotes Malignant Progression of Multiple Myeloma by Targeting TIMP2
Source: Front Cell Dev Biol. 2022 Mar 1;10:862524. doi: 10.3389/fcell.2022.862524 (PMC8921260; doi:10.3389/fcell.2022.862524)

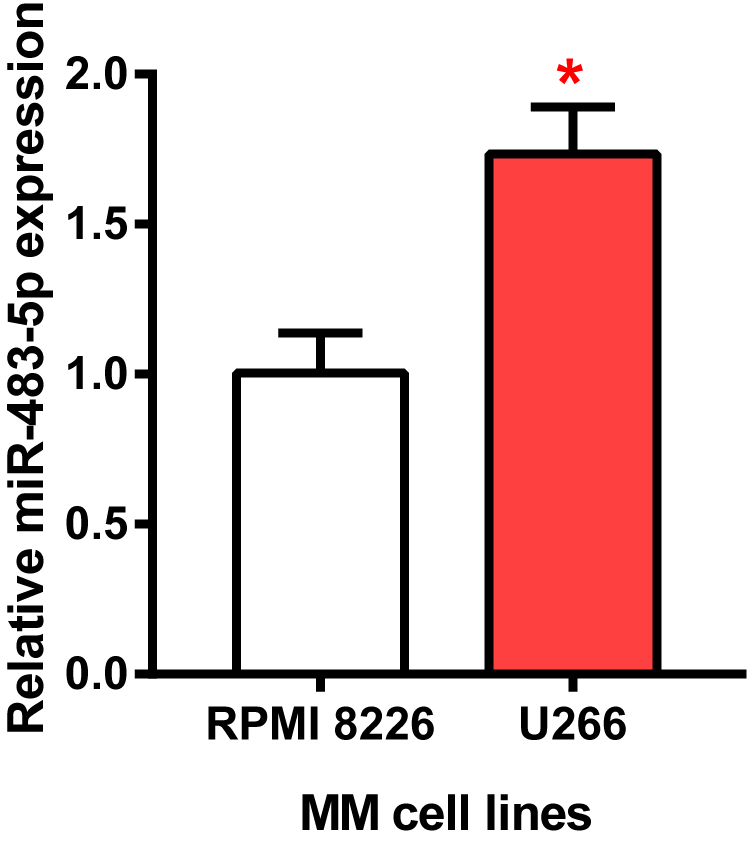

Supplement: Supplementary file 1 [file Image1.tif]
